# TBL1XR1 Promotes Coronary Artery Disease by Regulating Triglyceride Metabolism via the PPAR Pathway

**DOI:** 10.1155/crp/8877293

**Published:** 2025-12-28

**Authors:** Liping Yang, Liyuan Tang, Wenping Gao, Shanshan Zhu, Jiandi Liu, Yuhui He, Fanbo Meng

**Affiliations:** ^1^ Department of Geriatric Medicine, The Second People’s Hospital of Futian District, Shenzhen, 518049, Guangdong, China; ^2^ Department of Cardiology, The China-Japan Union Hospital of Jilin University, Changchun, 130033, Jilin, China, jlu.edu.cn

**Keywords:** coronary artery disease, PPAR pathway, *TBL1XR1*, triglyceride

## Abstract

**Introduction:**

Transducin beta‐like 1 X‐linked receptor 1 (TBL1XR1) is significantly upregulated in the peripheral blood of patients with coronary artery disease (CAD). This study aimed to validate the differential expression of TBL1XR1 in CAD and investigate its role in CAD progression using RNA interference.

**Methods:**

The expression of TBL1XR1 at the mRNA and protein levels was detected in patients with CAD and controls using reverse transcription–polymerase chain reaction (RT–PCR) and western blotting. Additionally, the effects of *TBL1XR1* gene silencing in human liver cells through RNA interference on PPARα expression and intracellular triglyceride (TG) levels were determined.

**Results:**

TBL1XR1 expression was significantly higher in the peripheral blood of CAD patients compared to controls at both mRNA (1.71 ± 0.96 vs. 1.00 ± 0.34, *p* < 0.01) and protein levels (0.41 ± 0.19 vs. 0.13 ± 0.07, *p* = 0.038). Logistic regression analysis revealed that high TBL1XR1 expression is an independent risk factor for CAD. Relative TBL1XR1 expression positively correlated with serum TG levels (rs = 0.56, *p* < 0.01) and Gensini score (rs = 0.53, *p* < 0.01), indicating an association with CAD severity. In human liver cells, TBL1XR1 silencing significantly increased peroxisome proliferator‐activated receptor alpha (PPARα) expression at both mRNA (*p* < 0.05) and protein levels (*p* < 0.01) while reducing intracellular TG levels (0.24 ± 0.16 vs. 0.51 ± 0.09, *p* < 0.01).

**Conclusion:**

*TBL1XR1* is a key factor for risk assessment, diagnosis, and evaluating coronary lesion severity in patients with CAD. Its role in promoting atherosclerosis initiation and development may be associated with regulation of TG metabolism via the PPARα pathway.

## 1. Introduction

Coronary artery disease (CAD) is one of the three leading causes of cardiovascular‐related deaths in China [1], imposing a substantial health burden and significant economic pressure. Therefore, identifying accurate and feasible methods for assessing CAD risk has become a pressing challenge.

Strong familial aggregation of CAD underscores the critical role of genetic factors in its onset. With advancements in gene chip technology, we have gained deeper insights into the molecular mechanisms underlying cardiovascular diseases [2, 3]. Whole blood gene expression analyses have been used to explore the complex mechanisms underlying cardiovascular disease, aiming to discover precise diagnostic methods and early risk markers for precision medicine [4–6]. Extensive research supports the value of peripheral blood gene expression analyses for assessing the risk of CAD [7–9] and monitoring the severity of coronary artery lesions and disease progression [10, 11], thereby contributing to the early prevention of cardiovascular events.

Using peripheral blood gene expression profiling, we previously identified 559 genes with significant differential expression between patients with CAD and controls [12, 13]. Among these, transducin beta‐like 1 X‐linked receptor 1 (*TBL1XR1*) was notably upregulated in CAD (LogFC = 1.710. *t* = 2.66, *p* = 0.025). Although TBL1XR1 has been implicated in cancer in bioinformatics analyses [14], its expression and roles in cardiovascular disease, particularly in CAD, remain unexplored.

To gain an in‐depth understanding of the function of *TBL1XR1* and its mechanism of action under physiological and pathological conditions, we searched the Reactome database (https://reactome.org). The results showed that *TBL1XR1* is involved in the regulation of lipid metabolism through the peroxisome proliferator‐activated receptor (PPAR) pathway. Abnormal lipid metabolism is one of the important risk factors for CAD; therefore, TBL1XR1 may indirectly affect the occurrence and development of CAD by regulating lipid metabolism. For this reason, in the present study, we aimed to further verify the differential expression of TBL1XR1 in the peripheral blood of patients with CAD, combine the results with clinical data, and analyze and discuss the reference significance of changes in the expression level of TBL1XR1 in peripheral blood for assessing the risk of CAD. In addition, RNA interference technology was used to explore the possible mechanism through which TBL1XR1 regulates triglyceride (TG) metabolism through the PPAR pathway, thereby promoting the occurrence and development of CAD.

## 2. Methods

### 2.1. Study Subjects

In this study, 96 patients with CAD who were hospitalized in the Department of Cardiovascular Medicine between October 2020 and October 2023 were randomly enrolled in the CAD group. The diagnosis of CAD followed the “Nomenclature and Diagnostic Criteria of Ischemic Heart Disease,” reported by the World Health Organization (WHO) in 1979. The extent of coronary atherosclerosis and stenosis was evaluated using imaging techniques, including percutaneous coronary angiography and coronary CT angiography (CTA), followed by quantification using the Gensini score. Higher scores indicate more significant atherosclerotic lesions. The control group consisted of 98 randomly selected individuals without CAD, and the absence of notable atherosclerotic lesions was confirmed using percutaneous coronary angiography or coronary CTA. The exclusion criteria were: (1) myocardial damage caused by reasons other than coronary atherosclerotic lesions; (2) severe heart failure, various cardiomyopathies, acute pulmonary embolism, or severe liver and kidney dysfunction; (3) presence of severe infections or unstable vital signs; and (4) failure to provide informed consent or incomplete clinical or imaging data.

Data collection included sex, age, body mass index (BMI), smoking history, family history of early‐onset CAD, and presence of other chronic conditions. Laboratory indicators included fasting blood glucose (FBG) and lipid profiles such as plasma TG, total cholesterol (TC), and low‐density lipoprotein cholesterol (LDL‐C).

### 2.2. Cells and Reagents

Human liver cells (L‐02, iCell‐h054) were purchased from iCell Bioscience Inc. (Shanghai, China). Complete RPMI‐1640 medium (KGM31800S) was obtained from KeyGEN BioTECH Co., Ltd. (Jiangsu, China). Incomplete RPMI‐1640 medium (61870‐036), OPTI‐MEM reduced serum medium (31985‐062), Lipofectamine 3000 transfection reagent (L3000015), and fetal bovine serum (FBS, 10099‐141) were purchased from Thermo Fisher Scientific Ltd. (Waltham, MA, USA). Penicillin–streptomycin (PS) solution (P1400) was provided by Solarbio Science & Technology Co., Ltd. (Beijing, China). The Total RNA Extraction Kit (RNAsimple Total RNA Kit) was obtained from Tiangen Biotech Co., Ltd. (Beijing, China). The Reverse Transcription Kit (TOYOBO ReverTra Ace qPCR RT kit) was obtained from TOYOBO Biotech Co., Ltd. (Shanghai, China). SYBR Premix Ex Taq Kit for qPCR was purchased from Takara Bio Co., Ltd. (Dalian, China). Peripheral blood lymphocyte separation medium was purchased from Haoyang Biological Products Technology Co., Ltd. (Tianjin, China). RIPA lysis buffer was purchased from Beyotime Biotechnology Co., Ltd. (Shanghai, China). The BCA Protein Assay Kit was purchased from Elabscience Biotechnology Co., Ltd. (Wuhan, China). The primary antibody for the internal control used in this study was Mouse Anti‐β‐Actin (HC201, 1:2000), obtained from TransGen Biotech Co., Ltd. (Beijing, China). Horseradish peroxidase (HRP)–conjugated Goat Anti‐Mouse IgG (*H* + *L*; GB23301, 1:2000), purchased from Servicebio Co., Ltd. (Wuhan, China), was used as a secondary antibody. Primary antibodies for the target proteins were Rabbit Anti‐TBL1XR1 (A7834, 1:1000) from Abclonal Biotechnology, Ltd. (Wuhan, China) and Rabbit Anti‐PPAR alpha (AF5301, 1:1000) from Affinity Biosciences Co., Ltd. (Jiangsu, China). HRP‐conjugated Goat anti‐Rabbit IgG (*H* + *L*; GB23303, 1:2000) was purchased from Servicebio Co., Ltd. The TG Assay Kit was obtained from Nanjing Jiancheng Bioengineering Institute. All reverse transcription–polymerase chain reaction (RT–PCR) primers were designed based on mRNA sequences (*TBL1XR1*, NM_024665.7; *PPARα*, NM_005036.6; *β-actin*, NM_001101.5) from the NCBI database (https://www.ncbi.nlm.nih.gov/) and were synthesized by General Biosystems Co., Ltd. (Anhui, China). Additionally, siRNA sequences were designed and constructed according to the *TBL1XR1* sequence, as shown in Table 1. Once the target sequences for the gene of interest were designed, they were synthesized by Tsingke Biotech Co., Ltd. (Beijing, China).

**Table 1:** Constructed siRNA sequences.

siRNA name	siRNA sequence (5′–3′)
TBL1XR1‐siRNA‐258	CGUAAUGCCUGAUGUAGUA(dT) (dT) UACUACAUCAGGCAUUACG(dT) (dT)
TBL1XR1‐siRNA‐879	GGACGCACAUACUGGUGAA(dT) (dT) UUCACCAGUAUGUGCGUCC(dT) (dT)
TBL1XR1‐siRNA‐1034	GGACAUACGAAUGAAGUAA(dT) (dT) UUACUUCAUUCGUAUGUCC(dT) (dT)
siRNA NC	UUCUCCGAACGUGUCACGU(dT) (dT) ACGUGACACGUUCGGAGAA(dT) (dT)

*Note:* TBL1XR1, transducin beta‐like 1 X‐linked receptor 1.

Abbreviation: NC, negative control.

### 2.3. Research Methods

#### 2.3.1. Peripheral Blood Collection

Peripheral whole blood samples, each of volume 4 mL, were collected from each participant using EDTA anticoagulant tubes and stored at 4°C. Total RNA and protein were extracted within 2 h of sample collection.

#### 2.3.2. Cell Culture and Transfection

L‐02 cells were cultured at 37°C with 5% CO_2_ in medium containing 89% complete RPMI‐1640, 10% FBS, and 1% PS. Once cells reached 80%–90% confluence, they were passaged. For experiments, cells were evenly seeded in six‐well plates at a density of 2 × 10^5^ cells per well and incubated in an incubator.

Cells were divided into blank control, negative control (transfected with siRNA‐NC), and interference groups (three groups, transfected with siRNA‐258, siRNA‐879, and siRNA‐1034). When cell density reached 70%, transfection was performed in the negative control and interference groups. Specifically, the medium was replaced with 1 mL of serum‐free medium. Two sterile EP tubes were prepared, each containing 125 μL OPTI‐MEM‐reduced serum medium. One tube was mixed with 5 μL of Lipofectamine 3000, while the other was mixed with 12.5 μL of siRNA (dissolved in DEPC water at 125 μL/1 OD). After incubating the samples at room temperature for 5 min, the contents of both tubes were combined and incubated for another 15 min before being added to the corresponding wells in a six‐well plate. After 4–6 h, 1 mL of complete medium containing 20% FBS was added to each well. TBL1XR1 expression was analyzed 48 h posttransfection using RT–PCR and western blotting. The siRNA with the most effective interference (siRNA‐258) was selected for subsequent experiments.

#### 2.3.3. RT–PCR

Total RNA was extracted from peripheral whole blood samples and cultured L‐02 cells using a Total RNA Extraction Kit. The quality and concentration of the extracted RNA were assessed via 1.5% agarose gel electrophoresis and spectrophotometry (NanoDrop 2000; Thermo Fisher Scientific). The quality was sufficient if electrophoresis displayed clear bands for 28S, 18S, and 5S, with a 28S‐to‐18S intensity ratio of 2:1, and the OD260/OD280 ratio was between 1.9 and 2.1. Subsequently, 1 μg of RNA was reverse‐transcribed using a reverse transcription kit, and PCR amplification was performed using a SYBR Green qPCR kit. Amplification was performed using a fluorescent PCR machine (CFX Connect; Bio‐Rad, Shanghai, China). The amplification conditions were as follows: predenaturation at 95°C for 10 min, 40 cycles of denaturation at 95°C for 10 s, annealing at 58°C for 30 s, and extension at 72°C for 30 s *β*‐Actin was used as the internal control gene, and the 2^−ΔΔCt^ method was employed for statistical analyses. The primer sequences are listed in Table 2.

**Table 2:** Primer sequences.

Gene name	Primer sequence (5′‐3′)
β‐actin F	AGGGAAATCGTGCGTGAC
β‐actin R	TAC​GGC​ATC​TAT​CAG​GGA​CAG
TBL1XR1 F	CAC​CCG​CTG​CAT​TGA​TTT​CTA
TBL1XR1 R	TAC​GGC​ATC​TAT​CAG​GGA​CAG
PPARα F	CCA​TCG​GCG​AGG​ATA​GTT​CT
PPARα R	GTG​AAA​GCG​TGT​CCG​TGA​TG

*Note: F*, forward; *R*, reverse.

#### 2.3.4. Western Blotting

Peripheral whole blood was subjected to mononuclear cell separation using human peripheral blood lymphocyte separation medium. RIPA lysis buffer was used to lyse peripheral blood mononuclear cells and cultured L‐02 cells, respectively, for total protein extraction. Protein concentrations were determined using the BCA Protein Assay Kit and adjusted to equal levels. Next, 30 μg of protein per sample was separated using SDS–PAGE, with settings of 60 V for protein stacking and 80 V for separation. Proteins were transferred onto membranes using a constant current of 300 mA for 90 min. Subsequently, the membranes were blocked at room temperature for 1 h with blocking buffer (TBS/0.1% Tween‐20 with 5% skim milk) and incubated overnight with primary antibodies. After washing, membranes were incubated with secondary antibodies at 4°C for 2 h. Detection and imaging were performed using an automatic chemiluminescence imaging system (Tanon‐5200; Tanon Science & Technology, Shanghai, China).

#### 2.3.5. TG Content Determination

Cells were divided into a blank control group, model group (50% FBS medium), negative control group (transfected with siRNA‐NC, 50% FBS medium), and SI‐TBL1XR1‐258 group (transfected with TBL1XR1 siRNA‐258, 50% FBS medium). After 48 h of transfection, the blank control group remained untreated, whereas the media of the model, negative control, and SI‐TBL1XR1‐258 groups were replaced with medium containing 50% FBS for further culturing. After 48 h, the medium was removed from the adherent cells, and the cells were rinsed twice with physiological saline. A small volume of liquid was retained in the bottle, and the cells were scraped using a cell scraper and transferred to centrifuge tubes. The cells were centrifuged at 1000 rpm for 10 min, and cell pellets were collected. These cells were disrupted using ultrasound at 300 W in an ice water bath for 3–5 s per burst, repeated four times with 30‐s intervals between bursts. TG levels were measured using the TG assay kit protocol. The optical density (OD) of each sample was measured at 562 nm using an automatic microplate reader (WD‐2012B; Liuyi Biotechnology Co., Ltd., Beijing, China). The TG concentration in each sample was calculated using a standard curve.

#### 2.3.6. Statistical Analysis

All data were analyzed using SPSS 22.0. Normally distributed measurement data are presented as the mean ± standard deviation. Independent *t*‐tests were conducted for pairwise comparisons, and one‐way analysis of variance (ANOVA) was used for comparisons among multiple groups. Alternatively, for non‐normally distributed data, interquartile ranges were used for statistical description, and between‐group differences were assessed using rank‐sum tests. Categorical data were described using frequency counts, and differences were analyzed using *χ* ^2^ tests. Risk factors associated with CAD were evaluated using binary logistic regression. Correlations between the relative expression levels of TBL1XR1, various indicators, and atherosclerosis severity were assessed using bivariate correlation analysis. Receiver operating characteristic (ROC) curves were generated based on the relative expression of TBL1XR1. Statistical significance was defined as a two‐tailed *p*‐value < 0.05.

## 3. Results

### 3.1. TBL1XR1 Expression in Peripheral Blood and Clinical Data

#### 3.1.1. Analyses of Clinical and Demographic Factors

The CAD and control groups did not differ significantly in terms of sex, age, BMI, TC, LDL‐C, family history of early‐onset CAD, smoking history, or presence of hypertension and diabetes. FBG and TG levels were significantly higher in the CAD group than in the control group, whereas HDL‐C levels were substantially lower in the CAD group (Table 3).

**Table 3:** Clinical data comparison between the CAD and control groups.

Data	CAD group (*n* = 96)	Control group (*n* = 98)	*t/* *χ* ^2^	*p*‐values
Age (year)	60.83 ± 10.32	58.53 ± 10.22	1.56	0.12
Sex			0.61	0.44
Male	63 (65.63)	59 (60.20)		
Female	33 (34.37)	39 (39.80)		
BMI (Kg/*m* ^2^)	24.94 ± 3.78	24.73 ± 3.58	0.39	0.70
Hypertension	45 (46.88)	43 (43.88)	0.18	0.67
Diabetes	19 (19.79)	12 (12.25)	2.06	0.15
Family history	13 (13.54)	8 (8.16)	1.45	0.22
Smoking history	50 (52.08)	41 (41.84)	2.04	0.15
FBG (mmol/L)	7.06 ± 2.92	5.70 ± 1.64	3.97	< 0.01^a^
TC (mmol/L)	4.57 ± 1.16	4.46 ± 1.04	0.66	0.51
TG (mmol/L)	2.23 ± 1.03	1.72 ± 0.91	2.12	0.03^a^
LDL‐C (mmol/L)	3.05 ± 0.99	2.89 ± 0.76	1.18	0.24
HDL‐C (mmol/L)	0.89 ± 0.21	1.07 ± 0.26	−5.03	< 0.01^a^

*Note:* Data are presented as the mean ± standard deviation or as *n* (%); TG, triglycerides.

Abbreviations: BMI, body mass index, CAD, coronary artery disease; FBG, fasting blood glucose; HDL‐C, high‐density lipoprotein cholesterol; LDL‐C, low‐density lipoprotein cholesterol; TC, total cholesterol.

^a^ *p* < 0.05.

### 3.2. RT–PCR Analysis of *TBL1XR1* Expression in Peripheral Blood

**Figure 1:**
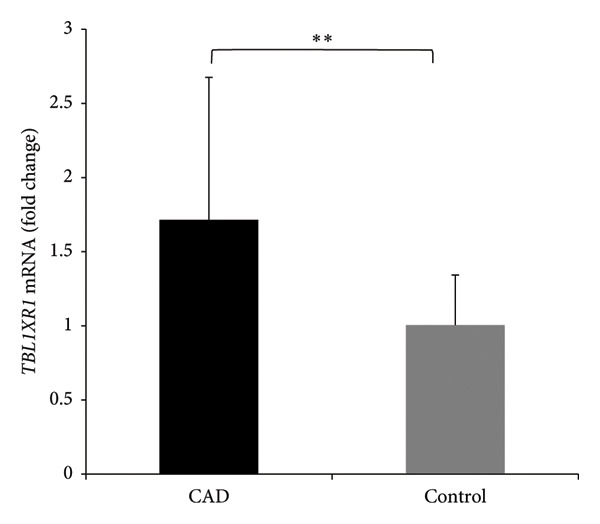
mRNA expression of *TBL1XR1* in peripheral blood. ^∗∗^ *p* < 0.01. CAD, coronary artery disease group; control, control group; *TBL1XR1*, transducin beta‐like 1 X‐linked receptor 1.

In RT–PCR analysis, the melting curve for the *TBL1XR1* gene exhibited a single peak, indicating the specificity of the amplified product. Additionally, the amplification curve demonstrated an “*S*” shape. Electrophoresis of the PCR products revealed a distinct bright band of 128 bp. Using the 2^−ΔΔCt^ method, the relative expression of *TBL1XR1* at the mRNA level was significantly higher in the CAD group than it was in the control group (1.71 ± 0.96 vs. 1.00 ± 0.34; *t* = 4.65, *p* < 0.01; Figure 1).

### 3.3. Western Blot Analysis of *TBL1XR1* Expression in Peripheral Blood

**Figure 2:**
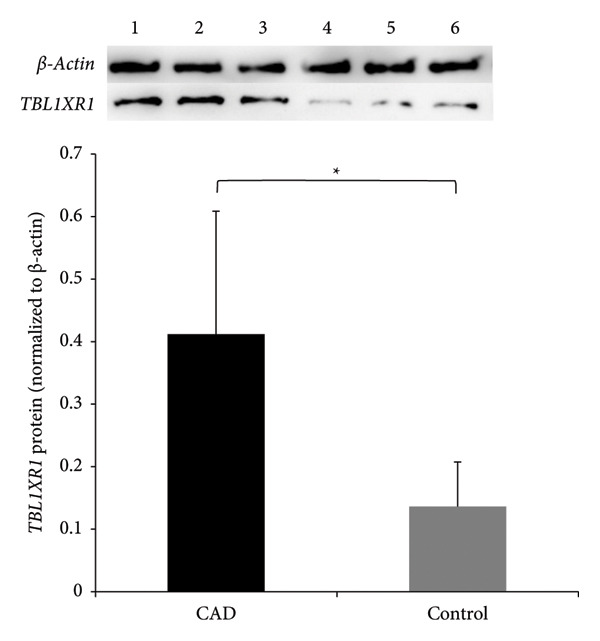
Western blot analysis of TBL1XR1 in peripheral blood. ^∗^ *p* < 0.05. CAD, coronary artery disease group (samples 1, 2, and 3); control, control group (samples 4, 5, and 6); TBL1XR1, transducin beta‐like 1 X‐linked receptor 1.

Using β‐actin as an internal reference gene, western blotting demonstrated that the protein expression level of *TBL1XR1* in the CAD group was notably higher than that in the control group (0.41 ± 0.19 vs. 0.13 ± 0.07, *t* = 2.65, *p* = 0.038; Figure 2).

### 3.4. Binary Logistic Regression Analysis of Risk Factors for CAD

In this study, Model 1 was constructed with “CAD status” as the dependent variable (coded as follows: 0 = non‐CAD and 1 = CAD) and general data (age; smoking history; and FBG, TC, TG, LDL‐C, and high‐density lipoprotein cholesterol [HDL‐C] levels) as independent variables. According to the median 2^−ΔCT^ value of *TBL1XR1* (0.079), 194 study participants were categorized into low‐*TBL1XR1* (2^−ΔCT^ ≤ 0.079) and high‐*TBL1XR1* expression groups (2^−ΔCT^ > 0.079). On the basis of Model 1, Model 2 was further established by incorporating the relative expression level of TBL1XR1 as an additional independent variable. The results are presented in Table 4. High *TBL1XR1* expression, advanced age, smoking history, elevated FBG level, and reduced HDL‐C level were closely associated with the onset of CAD and identified as independent risk factors for CAD. It is noteworthy that the upregulated expression of *TBL1XR1* increased the risk of developing CAD by 3.448‐fold. Moreover, the inclusion of the relative expression level of *TBL1XR1* enhanced the explanatory power of the model by 6.3%.

**Table 4:** Logistic regression analysis indicating independent risk factors of CAD.

Variables	Model 1		Model 2		
	*β*	*p*	*β*	*p*	OR (95%CI)
Age	0.05	0.01	0.044	0.013	1.045 (1.009–1.083)
Smoking history (1)	0.827	0.036	0.948	0.008	2.581 (1.277–5.214)
FBG	0.314	0.003	0.270	0.001	1.309 (1.114–1.540)
HDL‐C	−3.632	0.000	−2.370	0.007	0.093 (0.017–0.520)
Relative expression level of TBL1XR1(1)			1.238	0.001	3.448 (1.669–7.124)

*Note:* Coding criteria: smoking history (0 = non‐smoker, 1 = smoker); relative expression level of TBL1XR1 (0 = low expression, 1 = high expression). For Model 1: Nagelkerke *R* ^2^ = 0.325; Hosmer–Lemeshow test, *p* = 0.938. For Model 2: Nagelkerke *R* ^2^ = 0.388; Hosmer–Lemeshow test, p=0.115; TBL1XR1, transducin beta‐like 1 X‐linked receptor 1.

Abbreviations: CAD, coronary artery disease; CI, confidence interval; FBG, fasting blood glucose; HDL‐C, high‐density lipoprotein cholesterol; OR, odds ratio.

#### 3.4.1. Correlations Between *TBL1XR1* Expression and Biochemical Indicators

**Figure 3:**
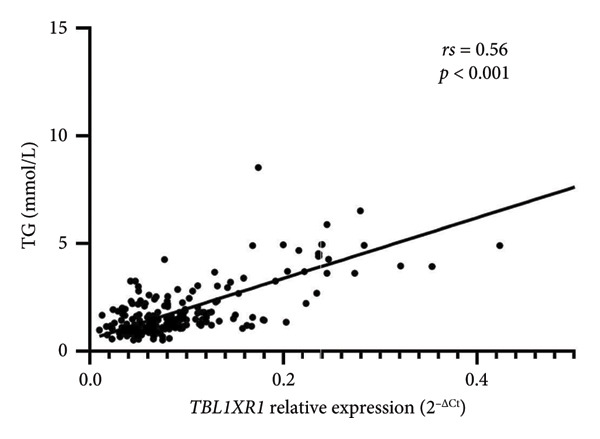
Correlation between the triglyceride (TG) levels and relative expression level of TBL1XR1.

Bivariate correlation analyses indicated a significant positive correlation between the relative expression of *TBL1XR1* and TG levels (*rs* = 0.56, *p* < 0.01). This result suggests that higher expression of *TBL1XR1* corresponds to increased plasma TG levels (Figure 3). Conversely, no significant correlations were observed between the expression of *TBL1XR1* and FBG, TC, or LDL‐C levels.

### 3.5. Relationship Between Relative TBL1XR1 Expression and Coronary Atherosclerosis Severity

**Figure 4:**
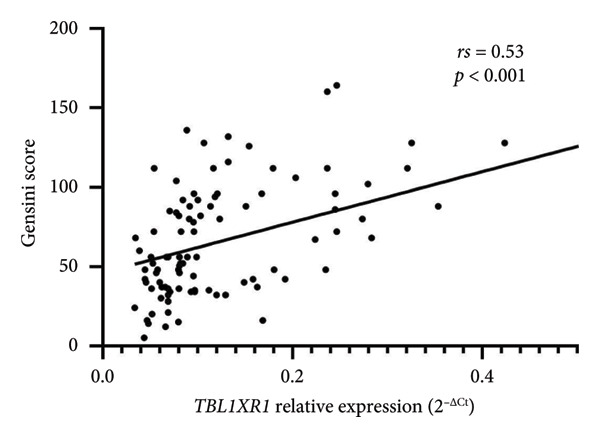
Correlation between the Gensini score and relative expression level of TBL1XR1.

The severity of coronary atherosclerosis in the CAD group was assessed using the Gensini score based on imaging results, with higher scores indicating greater severity. The Gensini score in the CAD group was 66.51 ± 15.41. Bivariate correlation analysis revealed a positive relationship between the relative expression of *TBL1XR1* in the peripheral blood and Gensini score (*rs* = 0.53, *p* < 0.01). This finding suggests that higher *TBL1XR1* expression in peripheral blood is associated with more severe coronary atherosclerosis (Figure 4).

### 3.6. ROC Curve Analysis of Relative *TBL1XR1* Expression

**Figure 5:**
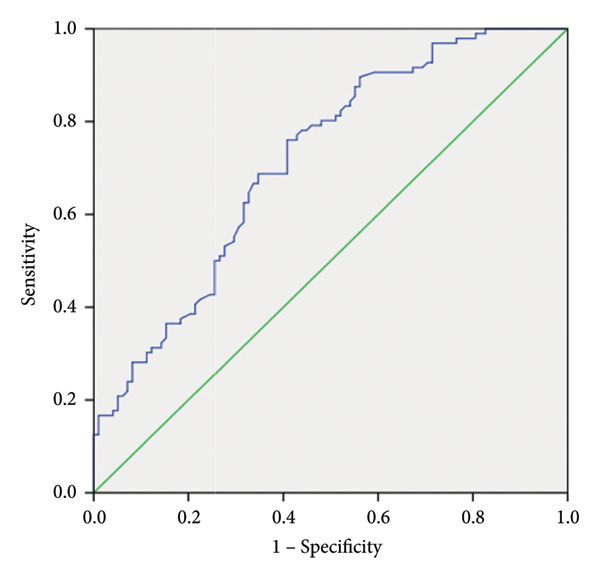
Receiver operating characteristic curve of the relative level of expression of *TBL1XR1.*

ROC curves were plotted based on the 2^−ΔCT^ values for relative *TBL1XR1* expression (Figure 5). High *TBL1XR1* expression in peripheral blood was a significant predictor for CAD, with an area under the curve (AUC) of 0.72 ± 0.04 (*p* < 0.01). The cutoff value, determined by the Youden index, was 0.068, yielding a sensitivity of 76.04% and a specificity of 59.18%.

### 3.7. Impact of TBL1XR1 Silencing on TG Metabolism via the PPARα Pathway

siRNA Transfection Efficiency.

**Figure 6:**
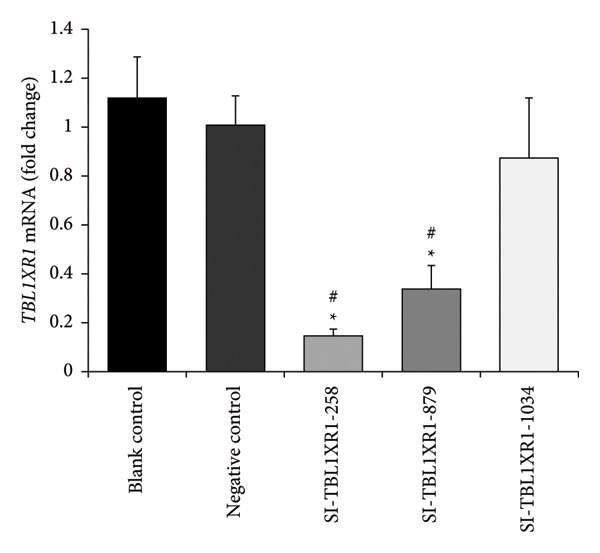
mRNA expression of *TBL1XR1* after transfection. #*p* < 0.01 vs. blank control group; ^∗^ *p* < 0.01 vs. negative control group; *TBL1XR1*, transducin beta‐like 1 X‐linked receptor 1.

**Figure 7:**
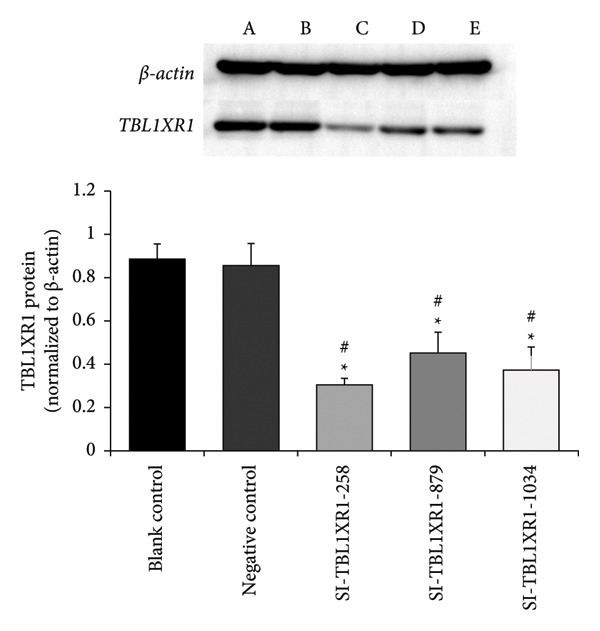
Western blot analysis of TBL1XR1 after transfection. #*p* < 0.01 vs. blank control group; ^∗^ *p* < 0.01 vs. negative control group; TBL1XR1, transducin beta‐like 1 X‐linked receptor 1.

At 48 h post‐transfection, RT–PCR results showed that the expression levels of *TBL1XR1* were significantly lower in the SI‐TBL1XR1‐258 group (0.15 ± 0.03) and SI‐TBL1XR1‐879 group (0.34 ± 0.09) compared with those in the blank control group (1.11 ± 0.17) and negative control group (1.01 ± 0.12; *p* < 0.01). Efficient interference was observed in the SI‐TBL1XR1‐258 group (Figure 6). Western blotting further confirmed that the protein expression of TBL1XR1 in the SI‐TBL1XR1‐258 (0.30 ± 0.04), SI‐TBL1XR1‐879 (0.45 ± 0.09), and SI‐TBL1XR1‐1034 groups (0.37 ± 0.11) was markedly lower than that in the blank control (0.88 ± 0.07) and negative control groups (0.85 ± 0.11; *p* < 0.01; Figure 7). Based on these results, siRNA‐258 was selected for subsequent experiments.

### 3.8. Changes in PPARα Expression Following Targeted *TBL1XR1* Silencing

**Figure 8:**
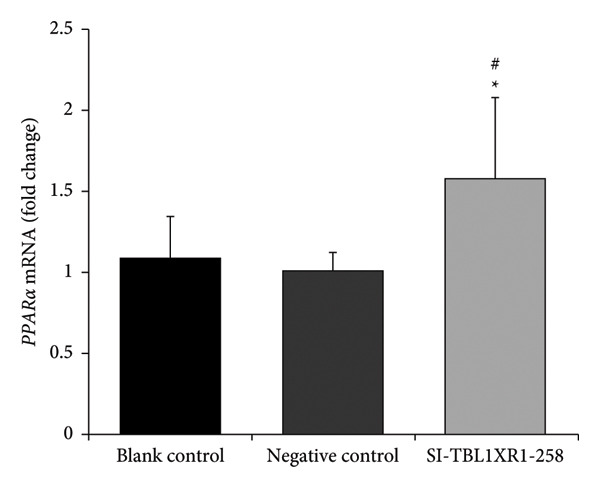
Relative expression of *PPARα* mRNA. #*p* < 0.05 vs. blank control group; ^∗^ *p* < 0.01 vs. negative control group; *PPARα*, peroxisome proliferator‐activated receptor alpha.

**Figure 9:**
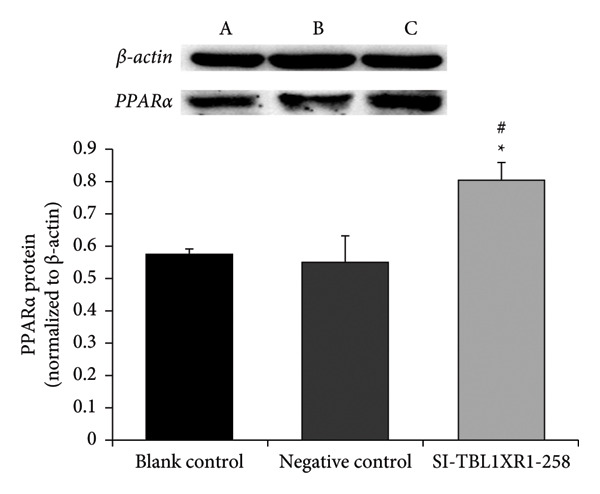
Western blot analysis of PPARα. #*p* < 0.01 vs. blank control group; ^∗^ *p* < 0.01 vs. negative control group; PPARα, peroxisome proliferator‐activated receptor alpha; sample A, blank control group; sample B, negative control group; sample C, SI‐TBL1XR1‐258 group.

Cultured cells were divided into blank control, negative control (transfected with siRNA‐NC), and SI‐TBL1XR1‐258 groups (transfected with TBL1XR1 siRNA‐258). At 48 h post‐transfection, RT–PCR and western blotting were performed to assess PPARα expression. The relative mRNA expression of *PPARα* was significantly higher in the SI‐TBL1XR1‐258 group (1.58 ± 0.13) than in the blank control (1.09 ± 0.25; *p* = 0.014) and negative control groups (1.00 ± 0.11; *p* = 0.007; Figure 8). Similarly, the protein expression of PPARα was higher in the SI‐TBL1XR1‐258 group (0.80 ± 0.05) than in the blank control (0.57 ± 0.02) and negative control (0.55 ± 0.08) groups (*p* < 0.01 for both comparisons; Figure 9).

### 3.9. TG Levels in Hepatocytes After *TBL1XR1* Silencing

**Figure 10:**
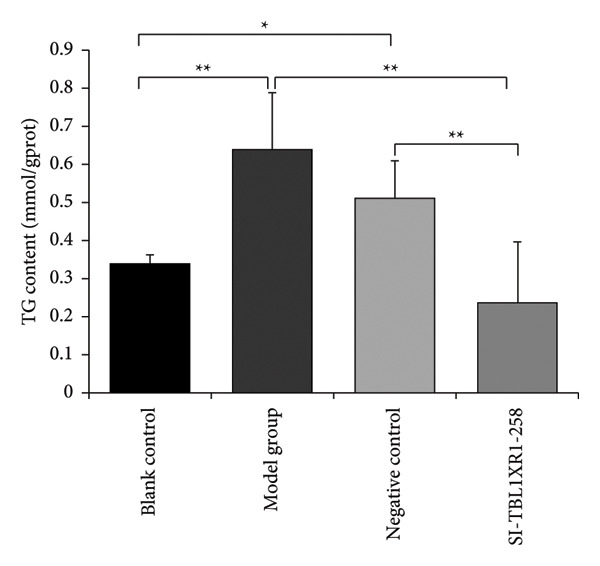
TG levels in hepatocytes after *TBL1XR1* silencing. ^∗^ *p* < 0.05; ^∗∗^ *p* < 0.01; TG, triglycerides.

At 48 h after transfection, the blank control group received no treatment, while the medium for the model, negative control, and SI‐TBL1XR1‐258 groups was switched to a culture medium containing 50% FBS for another 48 h of culture before TG levels were determined in each group. Following the medium change, TG levels in the model group (0.64 ± 0.15) and negative control group (0.51 ± 0.09) were significantly higher than those in the blank control group (0.34 ± 0.03; *p* < 0.05). In contrast, TG levels in the SI‐TBL1XR1‐258 group (0.24 ± 0.16) did not increase and were substantially lower than those in both the model and negative control groups (*p* < 0.01). Furthermore, there was no significant difference in the TG levels between the SI‐TBL1XR1‐258 and blank control groups (Figure 10).

## 4. Discussion

TBL1XR1 is a key transcriptional regulator, characterized by an F‐box/WD40 repeat structure. It consists of 18 exons and shares high homology with transducin beta‐like protein 1 (TBL1) [15, 16]. *TBL1XR1* was first identified in the human CD34+CD38− cell population. It has since been associated with tumor processes, including tumorigenesis, invasion, metastasis, and chemotherapy resistance [17–21]. TBL1XR1 is considered an important prognostic marker in oncology. Despite its well‐documented role in cancer, the involvement of TBL1XR1 in cardiovascular disease has not been determined. We previously showed that TBL1XR1 levels are elevated in the peripheral blood of patients with CAD. Building on these initial findings, the present study further validated the substantial elevation of TBL1XR1 expression at both the mRNA and protein levels in the peripheral blood of patients with CAD.

The logistic regression analyses showed that upregulated *TBL1XR1* expression in peripheral blood increased the risk of CAD by 3.448‐fold compared with that in the low expression group. High *TBL1XR1* expression, age, smoking, elevated FBG levels, and reduced HDL‐C levels were identified as independent risk factors for CAD. Additionally, a positive correlation was observed between Gensini score and relative *TBL1XR1* expression, indicating that higher *TBL1XR1* levels were associated with more severe coronary atherosclerosis. ROC curve analysis demonstrated that relative *TBL1XR1* expression had good sensitivity for CAD diagnosis. Nevertheless, its specificity was only 59.18%, likely due to the complex interplay of multiple genes in the pathogenesis of CAD. Changes in a single gene may not fully reflect the intricate mechanisms underlying CAD. However, this study highlighted the potential value of *TBL1XR1* in the risk assessment, diagnosis, and evaluation of the severity of coronary lesions in CAD. As more susceptibility genes are discovered in the future, combined multi‐gene testing will further improve its clinical utility.

Previous studies have identified *TBL1XR1* as an important factor in the regulation of multiple signaling pathways. In this study, bioinformatic analyses suggested that *TBL1XR1* modulates lipid metabolism through the PPAR pathway. Disrupted lipid metabolism, including elevated cholesterol, TG, and LDL levels, is a key risk factor for CAD [22]. Prior research has indicated a strong association between TBL1 and elevated plasma TG levels and liver steatosis [23], and *TBL1XR1* is highly homologous to TBL1. In this study, a comparison of clinical parameters revealed significant differences in FBG, TG, and HDL‐C levels between the CAD and control groups. A clear positive relationship was observed between *TBL1XR1* expression and TG levels. Based on these findings, we hypothesized that *TBL1XR1* plays an essential role in lipid metabolism. To explore this further, RNA interference was performed to functionally validate the role of *TBL1XR1.*

*PPARs* are a superfamily of nuclear transcription factors comprising three subtypes: *PPARα, PPARβ,* and *PPARγ*. Each subtype is encoded by distinct genes and exhibits vastly different patterns of tissue expression. *PPARα*, which is primarily expressed in the liver, is a key regulator of lipid metabolism [24]. Its selective agonists (such as fibrates) are widely used in clinical settings to effectively lower TG levels. In this study, *PPARα* was selected as a target gene to assess the effects of *TBL1XR1* knockdown. Our findings showed that silencing *TBL1XR1* led to significant upregulation of PPARα at both the mRNA and protein levels. Subsequently, the concentration of FBS in the culture medium was increased to stimulate TG synthesis in hepatocytes. Notably, under identical culture conditions, cells in the SI‐TBL1XR1‐258 group exhibited no significant increase in TG levels. This finding indicated that *TBL1XR1* downregulation substantially attenuated intracellular TG accumulation. Consequently, *TBL1XR1* modulated TG metabolism by regulating *PPARα* expression, thereby contributing to the initiation and development of coronary atherosclerosis.

An ideal disease biomarker should be easily accessible, minimally invasive, and convenient to use. For this reason, peripheral blood was chosen for differential gene expression analyses, aiming to identify a reliable marker for CAD. Nevertheless, further investigation is warranted to determine whether the expression of *TBL1XR1* in peripheral blood aligns with that in liver cells.

## 5. Conclusions

*TBL1XR1* was significantly upregulated in the peripheral blood of patients with CAD, and elevated *TBL1XR1* expression was identified as an independent risk factor for CAD. Higher *TBL1XR1* expression was correlated with increased severity of coronary atherosclerosis. *TBL1XR1* may promote the initiation and development of coronary atherosclerosis by regulating TG metabolism via the PPAR pathway. These findings clarify the mechanisms by which *TBL1XR1* contributes to CAD and indicate that *TBL1XR1* may be valuable for the risk assessment, diagnosis, and evaluation of the severity of coronary lesions.

## Ethics Statement

The study protocol was reviewed and approved by the Ethics Committee of the Second People’s Hospital of Futian District, Shenzhen, China (approval number: 20230412YLP). All samples and information from participants were collected after obtaining written informed consent.

## Disclosure

The funder had no role in the design, data collection, data analysis, and reporting of this study.

## Conflicts of Interest

The authors declare no conflicts of interest.

## Author Contributions

Study design and manuscript​ review: Fanbo Meng, Liping Yang, Yuhui He, Liyuan Tang, Wenping Gao, Jiandi Liu, and Shanshan Zhu. Data collection, data analysis, and experimental implementation: Liyuan Tang, Wenping Gao, Jiandi Liu, and Shanshan Zhu. Manuscript preparation: Liping Yang, Liyuan Tang, and Wenping Gao. Critical revision of the manuscript for intellectual content: Fanbo Meng.

## Funding

This study was supported by the Futian Healthcare Research Project (FTWS2023078).

## Data Availability

The data that support the findings of this study are available from the corresponding author upon reasonable request.
